# Role of MYC-miR-29-B7-H3 in Medulloblastoma Growth and Angiogenesis

**DOI:** 10.3390/jcm8081158

**Published:** 2019-08-02

**Authors:** Ian J. Purvis, Janardhan Avilala, Maheedhara R. Guda, Sujatha Venkataraman, Rajeev Vibhakar, Andrew J. Tsung, Kiran K. Velpula, Swapna Asuthkar

**Affiliations:** 1 Departments of Cancer Biology and Pharmacology, University of Illinois College of Medicine Peoria, Peoria, IL 61605, USA; 2 Department of Pediatrics, University of Colorado School of Medicine, Aurora, CO 80045, USA; 3 Departments of Neurosurgery, University of Illinois College of Medicine Peoria, Peoria, IL 61605, USA; 4 Departments of Pediatrics, University of Illinois College of Medicine Peoria, Peoria, IL 61605, USA

**Keywords:** medulloblastoma, MYC, B7-H3, miR-29, p-STAT1, angiogenesis

## Abstract

Medulloblastoma (MB) is the most common embryonal neuroepithelial tumor, with poor patient outcomes and secondary complications. In this study, we investigated the role of the B7 family of immune checkpoint homolog 3 (B7-H3) expression in MB angiogenesis. B7-H3, a co-inhibitory immune checkpoint, is highly expressed and is associated with lower overall survival in MYC^+^ MB’s. Evidence for a direct transcriptional role of MYC on the *B7-H3* gene promoter was confirmed by MYC inhibition and anti-MYC antibody ChIP analysis. Interestingly, MYC inhibition not only downregulated the B7-H3 protein expression, but also rescued miR-29 expression, thus indicating a triangular regulatory relationship between MYC, miR-29, and B7-H3 in Group 3 MB cells. From RNA seq and IPAD assay, we observed a negative feedback loop between miR-29 and MYC that may control B7-H3 expression levels in MB cells. Our studies show that B7-H3 expression levels play a crucial role in promoting MB angiogenesis which can be inhibited by miR-29 overexpression via miR-29-mediated B7-H3 downregulation. The tumor suppressor role of miR-29 is mediated by the activation of JAK/STAT1 signaling that further plays a role in MYC-B7-H3 downregulation in MB. This study highlights B7-H3 as a viable target in MB angiogenesis, and that the expression of miR-29 can inhibit B7-H3 and sensitize MB cells to treatment with MYC-inhibiting drugs.

## 1. Introduction

Medulloblastoma (MB) is the most common embryonal neuroepithelial tumor [1], accounting for approximately 20% of all pediatric brain tumors. Standard therapy includes tumor resection and chemotherapy and/or craniospinal irradiation [2]. MB patients tend to incur secondary complications from treatment that includes stymied neurocognitive development, improper hormonal production, and temporary mutism among other sequelae [2]. The characterization of genetic and epigenetic alterations and methylation profiling has led to the useful classification of 4 distinct MB subgroups that contain evolving subtypes within each subgroup: WNT, SHH, Group 3 and Group 4 [2,3]. Recent efforts are underway to evaluate subgroup-specific therapies, but improved therapeutic targets will aid in the development of more effective treatments (Clinical Trial ID: NCT03434262). Group 3 and 4 Myc^+^ MB tumors tend to have a worse prognosis than Myc^-^ tumors, suggesting that Myc dysregulation leads to greater resistance to current treatment options [4]. Group 3 tumors tend to overexpress c-Myc (MYC), while Group 4 tumors tend to exhibit more frequent N-Myc dysregulation than MYC [4,5]. Additionally, MYC^+^ MB tumors tend to metastasize more frequently than other MB tumors [6]. Despite the negative outcomes associated with MYC amplifications, microRNAs hold promise as anti-tumor agents. One such microRNA, miR-29, was shown to be downregulated due to high MYC expression in both leukemia and lymphoma cells [7,8].

miR-29, a known tumor suppressor, is repressed in a variety of cancers, including osteosarcoma, lymphoma, and nasopharyngeal carcinoma. Recent literature has shown that restoration of miR-29 leads to anti-tumor effects, including decreased proliferation and migration and increased anti-tumor epigenetic modifications [9,10,11]. In addition, miR-29 restoration shows pro-apoptotic effects in glioblastoma by downregulating epithelial-mesenchymal transition molecules such as COL1A2, COL3A1, COL4A1, ELN, ITGA11 and MMP24. Further, Shin et al. also reported that miR-29 downregulated SPARC, a molecule that activates the PI3K/AKT pathway and tumor angiogenesis [12]. To date, the role of miR-29 and its relationship to MYC in MB has not been investigated.

B7-H3, an inhibitory immune checkpoint ligand, is highly expressed compared to other notable immune checkpoints, like PD-1/PD-L1 and CTLA-4, in MB tumor tissues and cell lines [13]. B7-H3 plays a critical role in modulating the tumor microenvironment, and is likely responsible for downregulating T cell function in various cancers [14]. Studies from the last decade indicate that B7-H3 could be a multimodal therapeutic target due to its role in tumor cell migration/invasion, immune evasion and metastasis [15,16,17,18]. Encouragingly, recent in vivo studies have shown B7-H3 to be a promising therapeutic target in medulloblastoma mouse models [19]. However, the comprehensive role of B7-H3, including its regulation and identification of its receptor(s), has yet to be fully elucidated. Additionally, the mechanistic role that B7-H3 plays in MB, as well as its potential as a therapeutic target, has not been fully determined.

Our studies indicate that B7-H3 is upregulated in MYC^+^ cells and promotes MB angiogenesis. We show that a miR-29 overexpression-based approach can be used to target B7-H3 in MB. Further, we provide evidence for a regulatory relationship between MYC-miR-29-B7-H3 in MB, and that targeting this regulatory loop inhibits the angiogenic abilities of MB cells and may sensitize them to MYC-targeted chemotherapeutic treatments.

## 2. Materials and Methods

### 2.1. Ethics Statement

All tissue specimens were obtained from the Children’s Hospital, OSF Saint Francis Medical Center (Peoria, IL, USA) and processed in accordance with the UICOMP Institutional Review Board-approved protocol (protocol # 491250-2, version dated 21 August 2013 and renewed on 14 May 2018). The 5 human MB tumor tissues used in this study were obtained from patients (Age: 2–16 years) undergoing therapeutic surgery. Normal cerebellum tissue samples were obtained from patient biopsies due to other non-disclosed conditions. All sample diagnoses (as tumor tissues or normal cerebellum tissue) and staging were confirmed by a pathologist.

### 2.2. In Silico Analysis

B7-H3 transcript in MB tissues was assessed by Oncomine (www.oncomine.org). Kaplan-Meier survival curve plotted using R2 database (http://r2.amc.nl). The ‘Upstream Regulator Analysis’ and ‘Mechanistic Networks’ are available within IPA tool (http://www.ingenuity.com) (see supplementary data).

### 2.3. Cell Culture and Transfection

The D283 Med (D283; RRID:CVCL_1155), D425 Med (D425; RRID:CVCL_1275) and D458 (D458; RRID:CVCL_1161) cells were grown in complete DMEM media (10% FBS, 1% penicillin/streptomycin, 1% Sodium Pyruvate) (ThermoFisher, Waltham, MA, USA). Cells were kindly gifted by Dr. Rajeev Vibhakar, University of Colorado and were authenticated using STR profiling (University of Colorado, Denver, CO, USA). HUVEC cells (Life Technologies, Carlsbad, CA, USA) were grown in Medium 200 (ThermoFisher) supplemented with low serum growth supplement (ThermoFisher). Cells were incubated at 37 °C with 5% CO_2_. Transfections were carried out using Lipofectamine 2000 (ThermoFisher) (see supplementary data). 

### 2.4. Plasmids, Antibodies, and Chemical Inhibitors

miR-29b/a plasmid was purchased from Addgene (Cambridge, MA, USA). Plasmids for shB7-H3 were purchased from Origene (Rockville, MD, USA). B7-H3 overexpression plasmid (B7-H3 OE) was purchased from Sino Biological Inc. (Wayne, PA, USA). A pCDNA3-scrambled vector was used as a control. Antibodies for B7-H3, c-Myc, p-STAT1, STAT1, IGF-1R, Cofilin, N-cadherin, CDK-6, Caspase 3, and Actin were purchased from Santa Cruz Biotechnology Inc (Dallas, TX, USA). shMYC siRNA was also purchased from Santa Cruz. HRP conjugated secondary antibodies were purchased from Novus Biologicals (Littleton, CO, USA). The MYC inhibitor, JQ1, was purchased from Cayman Chemicals (Ann Arbor, MI, USA). All-*trans* retinoic acid (ATRA) was purchased from Sigma Aldrich (St. Louis, MO, USA).

### 2.5. ELISA, Human Angiogenesis Array, Immunoblotting and Gelatin Zymography

Indirect ELISA to estimate the soluble form of B7-H3 and MMP-9 proteins was done following the standard protocol [20]. Human Angiogenesis Array C100 (RayBiotech, Peachtree Corners, GA, USA) was incubated with equal volumes of conditioned media from D283 control, miR-29-transfected or B7-H3 OE-transfected cells. Array incubation, washing and visualization were performed according to manufacturer instructions. Densitometry was carried out using ImageJ software. Densitometry normalization for comparison was calculated according to manufacturer recommendation. Immunoblot and MMP-2/9 gelatin zymography were performed as described previously [21]. Buffers and staining reagents were formulated according to Abcam’s protocol (https://www.abcam.com/protocols/gelatin-zymography-protocol).

### 2.6. F-actin Staining and Immunostaining

The FIT-phalloidin-based F-actin red fluorescence (Abcam, Cambridge, UK) staining was done on HUVEC cells alone or HUVEC co-cultured with D283 and D425 cells on 3-well slides (ibidi, Fitchburg, WI, USA). The cells were grown for 24 h, fixed and stained according to the vendor’s instructions. Calcein AM green fluorescence (Invitrogen, Carlsbad, CA, USA) was used to stain D283 and D425 cells in the co-culture experiment. The images were captured with an Olympus BX61 Fluoview confocal microscope (Olympus, Center Valley, PA, USA) at 40× magnification. The immunostaining detection of B7-H3 protein on the MB tissue sections and cells was done following a standard protocol [21].

### 2.7. Real-Time PCR and RNA-Seq

Total RNA was isolated using TRIZOL reagent (Invitrogen, Carlsbad, CA, USA). Real-time reactions were performed using CFX96 Real-Time System (Bio-Rad, Hercules, CA, USA) [21]. The RT-PCR specific primers are listed in Table S1. The RNA sSequencing was performed by Novogene (Chula Vista, CA, USA). Total RNA extracted was sequenced on an Illumina HiSeq 2000 platform with a paired-end 150 bp sequencing strategy. On average, 51.4 million reads were obtained per sample. Raw read of fastq format were then processed through Novogene in-house perl scripts to obtain clean reads (49.4 million on average), by removing reads containing adapters, reads containing poly-N and low-quality reads from raw data. Index of the reference genome was built using Bowtie v2.2.3 (Johns Hopkins University, Baltimore, MD, USA), and paired-end clean reads were aligned to the reference genome using TopHat v2.0.12. (Johns Hopkins University) HTSeq v0.6.1 (Open source, http://www-huber.embl.de/HTSeq) was used to count the reads numbers mapped to each gene; 87.17% of reads were uniquely mapped. FPKM (fragments per kilobase of transcript per million mapped reads) of each gene was calculated based on the length of the gene and reads count mapped to this gene. Differential expression analysis was performed using the DESeq R package (1.18.0) (Open source, Bioconductor.org). The *p*-values were adjusted using the Benjamini and Hochberg’s method. Genes with an adjusted *p*-value < 0.05 were considered as differentially expressed.

### 2.8. IPAD Analysis

Immuno-paired antibody detection (IPAD) (ActivSignal, Natick, MA, USA) assay to measure 70 key proteins involved in more than 20 signaling pathways (https://www.activsignal.com/service/) was done using D283 control and miR-29-treated cells. The paired oligo-tagged antibodies were quantified by digital PCR measured from a Fluidigm Biomark apparatus.

### 2.9. Chromatin Immunoprecipitation (ChIP) and DNA Sequencing

ChIP assay was performed using the ChIP-IT Express Kit from Active Motif, and DNA sequencing and MYC binding site prediction analysis were done following previously standardized protocol [22].

### 2.10. Fluorescence-Activated Cell Sorting (FACS) Analysis 

The percentages of treated cells in various cell cycle phases (G0/G1, S, and G2/M) were analyzed by FACS according to previously standardized methods [23].

### 2.11. In-Vitro Angiogenesis Assay

The angiogenesis assay was performed using 40,000 HUVECs incubated with conditioned media on 50 µL of matrigel (Corning Inc, Corning, NY, USA) coated 96-well plates. Images were taken with Olympus IX71 (4×) at time point of 4 h to visualize the difference in ring and tube formation. Branching points from HUVEC rings were manually assessed.

### 2.12. Chick Chorioallantoic Membrane Assay (CAM)

Fertilized white leghorn chicken eggs were purchased from Charles River Laboratories (Wilmington, MA, USA). The assay protocol was adapted from a previous standard protocol [24]. Briefly, small windows were made in the egg shells, and incubated at 37 °C with 50% humidity for 7 days. 40,000 cells suspended in serum-free matrigel solution (2 mg/mL) were then inoculated into the eggs, and incubated for 48 h. Images were obtained using an Olympus SZX12 Stereo Microscope. Angiogenesis in terms of the number/length of blood vessels formed was quantified using ImageJ v1.52p (National Institutes of Health, Bethesda, MD, USA)

### 2.13. Statistical Analysis

Statistical analysis and graphing were performed using Origin version 9.0 software (Microcal Software Inc., Northampton, MA, USA). Statistical significance was calculated using one-way analysis of variance (ANOVA), and data were expressed as mean ± S.E. In all figures, statistical significance is labeled the following way: *, *p* < 0.05; **, *p* < 0.01; ***, *p* < 0.001.

## 3. Results

### 3.1. High Expression of B7-H3 Is Associated with Poor Survival in MB Patients

**Figure 1:**
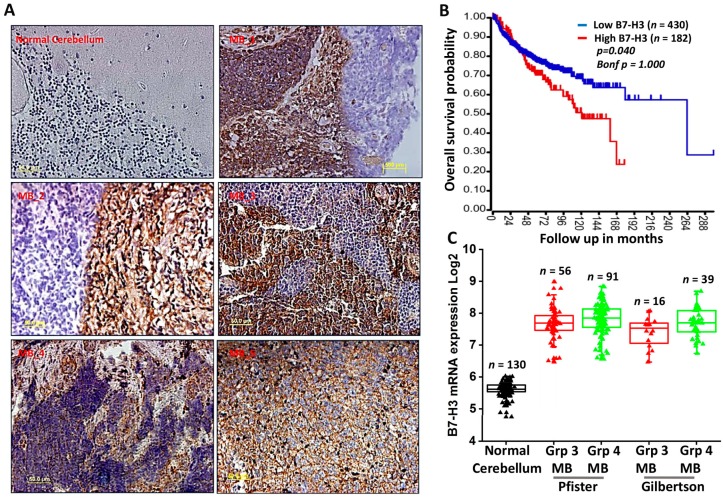
B7-H3 is elevated in MB patient samples and is associated with poorer survival. (**A**) Immunostained MB patient tissue samples compared to normal cerebellum against B7-H3 (**brown**) with nuclear staining (**blue**). B7-H3 expression detected in 5 MB tissue samples but not in normal cerebellum. (**B**) Kaplan-Meier survival plot showing overall survival probability of MB samples with high (**red**) or low (**blue**) B7-H3 mRNA expression in months (*p* = 0.040). Transcript data obtained from Cavalli dataset and analyzed by log rank test. (**C**) Box plots of B7-H3 mRNA levels across 2 MB subgroups from Northcott dataset plotted on Log2 scale.

B7-H3 is a known suppressive immune checkpoint that has increasingly been implicated in cancer progression [17,25]. To verify if B7-H3 could be considered a therapeutic target in MB, biopsy specimens obtained from human MB tissues were stained using a B7-H3 antibody. Immunohistochemical analysis performed on five MB specimens confirmed the expression of B7-H3 protein in tumor samples when compared to normal human cerebellum tissue (Figure 1A). To corroborate our findings, we verified the mRNA expression profile of B7-H3 and its association with overall survival of MB patients using datamining studies. Kaplan-Meier survival curves plotted using the Cavalli dataset available through the R2 software (http://r2.amc.nl) showed reduced patient survival with increased expression of B7-H3 (Figure 1B). Further, mRNA data from Pfister and Gilbertson datasets was obtained from the R2 software. Datamining studies across these patient datasets showed that B7-H3 is more prevalent in MB when compared to other important immune checkpoints such as PD-L1 and CTLA-4 (Supplementary Figure S1A). We also observed elevated expression of B7-H3 transcripts across the Group 3 and Group 4 subgroups of MB compared to normal cerebellar tissue (Figure 1C), indicating that a higher expression of B7-H3 is associated with shorter overall survival in aggressive types of MBs.

### 3.2. B7-H3 Can Be Regulated via Myc-miR-29 Axis in MB Cells

**Figure 2:**
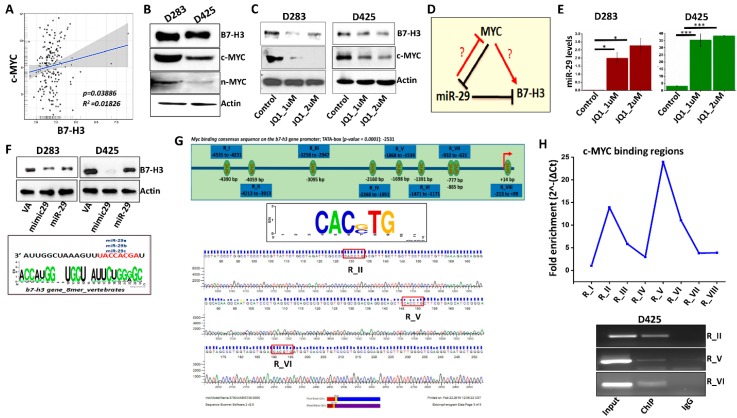
B7-H3 expression is regulated by Myc and miR-29. (**A**) mRNA transcript levels of Myc and B7-H3 in MB patient samples from Northcott dataset. (**B**) Protein expression levels of B7-H3, c-Myc and n-Myc in D283 and D425 cells determined by western blotting with Actin as a loading control. (**C**) Western blot analysis of c-Myc and B7-H3 protein levels in D283 and D425 cells treated with JQ1 (1 μM and 2 μM) incubated for 24 h. Actin used as loading control. (**D**) Schematic representation of Myc-miR-29-B7-H3 regulatory axis. (**E**) miR29 transcript levels in D283 (**left**) and D425 (**right**) cells treated with JQ1, 24 h incubation as determined by RT-PCR (*n* = 4 for each group). (**F**) Western blotting showing B7-H3 expression in D283 (**left**) and D425 (**right**) cells transfected with scrambled vector (VA), miR-29 mimic (mimic29) or miR-29b/a plasmid (miR-29). Cells were harvested 48 h after transfection, Actin used as loading control. (**Bottom**) Analysis of 3’ UTR of B7-H3 mRNA with consensus binding sequence homology with miR-29 isoforms. Developed from microRNA.org database. (**G**) B7-H3 promoter analysis showing 9 potential Myc-binding sites (CAC(GC)TG; *p =* 0.0001) on promoter sequence from −5000 to +100 bp. (**Top**); Promoter sequence developed from Eukaryotic Promoter Database. (**Bottom**); Sequencing analysis of RI, RV and RVI regions. (**H**) To investigate MYC binding to the *B7-H3* gene promoter, D425 DNA was immunoprecipitated with anti-MYC and anti-IgG antibody. ChIP DNA was analyzed by qRT-PCR using ChIP-specific primers spanning the regions, R-I to R-VIII (primers listed in Table S2), (**Top**); fold enrichment of MYC protein on the *B7-H3* promoter region (RI-RVIII) was quantified and represented graphically. (**Bottom**); agarose gel showing the PCR amplified regions (RI, RV and RVI) using input, IgG and ChIP DNA. * *p* < 0.05, ** *p* < 0.01, *** *p* < 0.001.

The MYC family of proteins are highly expressed in MBs [4]. The c-MYC protein is more intricately linked to Group 3 MBs, and its amplification is correlated with poor patient outcomes [3,4,6]. c-MYC withdrawal causes complete regression of Group 3 MB; however, targeting MYC alone did not yield favorable outcomes. Therefore, identifying bona-fide MYC–associated targets such as B7-H3 is needed. Further support for B7-H3 as a potential therapeutic target comes from its positive overall correlation with MYC using Northcott (*p* = 0.03668) and Cavalli (*p* = 1.33 × 10^−03^) MB datasets, suggesting the existence of a regulatory relationship in MB tumors (Figure 2A, Supplementary Figure S1B,C). Next, we began examining whether B7-H3 expression is associated with MYC in different MB cell lines. Both D283 and D425 cells showed higher expression of c-MYC than n-MYC at the protein and mRNA levels (Figure 2B and Supplementary Figure S1D). qRT-PCR analysis conducted on MB cells, D283 and D425, showed that MYC transcripts are higher in D283 than D425 (Supplementary Figure S1D). To further investigate the transcriptional relationship between MYC and B7-H3, D283 and D425 cells were treated with JQ1, a BET family bromodomain inhibitor that impairs MYC function [26]. IC50 values for JQ1 treatment were 1 µM as determined by MTT assay (Supplementary Figure S1E). Treatment of D283 and D425 cells with JQ1 showed a notable decrease in MYC and B7-H3 expression when compared to control cells (Figure 2C). Treatment of D283 cells with shMYC plasmid also showed a decrease in B7-H3 and MYC expression (Supplementary Figure S2A), but due to the clinical relevance of JQ1 [26,27], subsequent experiments were conducted using JQ1.

MYC forms a corepressor complex and binds to the miR-29 promoter, downregulating its expression [10]. Further, miR-29 was shown to directly target the 3′ untranslated region of B7-H3, and knockdown of miR-29 leads to an up-regulation of B7-H3 protein expression in various solid tumors [28]. These observations indicate that MYC can control the transcription of miR-29 and B7-H3 and suggests a triangular regulatory relationship between MYC, miR-29 and B7-H3 (Figure 2D). To date, this MYC-miR-29-B7-H3 regulatory relationship has not been verified in MB. From RT-PCR analysis, we observed that the endogenous levels of miR-29 were low in D283, D425, and D458 cells (Supplementary Figure S2B). However, inhibition of MYC using JQ1 elevated the miR-29 expression in both D283 and D425 cells indicating an inverse correlation between MYC and miR-29 in Group 3 MBs (Figure 2E). miR-29 overexpression was induced using a miR-29 mimic or plasmid in D283 and D425 cells, which showed a decrease in B7-H3 protein levels (Figure 2F). Transfection with shB7-H3 plasmid exhibited poorer inhibition of B7-H3 expression compared to miR-29 (Supplementary Figure S2C). As suggested by previous studies, miR-29 may be a more clinically useful therapeutic strategy for inhibiting B7-H3 [29]. Together, these results suggest that MYC^+^ MB cells regulate B7-H3 protein at least in part by repressing miR-29 levels.

### 3.3. MYC May Regulate B7-H3 Transcription

To date, no studies have investigated the regulatory mechanism between MYC and B7-H3 in MB. We observed 9 potential MYC binding sites (*p* > 0.0001) on the *B7-H3* promoter (Ref-Seq: NM_001329628) using the Eukaryotic Promoter Database (EPD). The *B7-H3* promoter sequence (−5000 bp to +100 bp) in humans contained conserved MYC binding sequences of CAC(G,C)TG across 4 species (Figure 2G, top panel). To determine which binding sites are most often targeted by MYC, ChIP DNA immunoprecipitated by anti-MYC and anti-IgG antibodies were scanned for the MYC binding regions (RI-RVIII) in the *B7-H3* promoter by qRT-PCR. The primers for regions (R) II, V and VI showed the highest amplification, indicating greater MYC enrichment at RII, RV, and RVI when compared to the other regions (I, III, IV, VII, VIII) (Figure 2H). The presence of MYC binding sites were then confirmed by sequencing of ChIP-amplified DNA samples using primers for RII, RV and RVI. (Figure 2G, lower panel). Collectively, these results support the hypothesis that MYC regulates B7-H3 transcription by directly binding to specific regions within the *B7-H3* promoter. 

### 3.4. B7-H3 Promotes Angiogenesis in MB Cells

**Figure 3:**
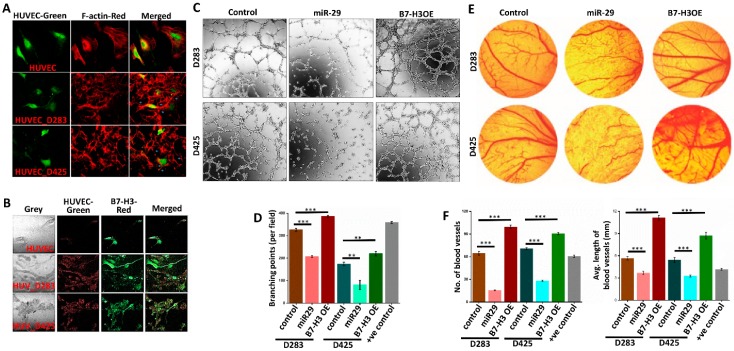
B7-H3 promotes and miR-29 inhibits angiogenesis of HUVEC cells. (**A**) Co-culture of HUVEC cells alone (**top**), with D283 cells (**middle**), or with D425 cells (**bottom**). HUVEC cells were stained with calcein AM (**green**). F-actin (**red**) staining shows actin filament networks. (**B**) Co-culture of HUVEC cells alone (**top**), with D283 cells (**middle**), or with D425 cells (**bottom**) showing B7-H3 expression levels. HUVEC’s were stained with calcein AM (**green**), and B7-H3 (**red**) was stained with AlexaFluor 594. (**C**) Angiogenesis assay of HUVEC cells incubated with D283 and D425 conditioned media for 4 h. HUVEC’s (40,000 cells/well) were plated onto Matrigel-coated (10 mg/mL) 96-well plates in 100 μL of control, miR-29 plasmid-transfected, or B7-H3 OE plasmid-transfected cells. (**D**) Branching points from HUVEC rings were manually assessed and averaged from 3 representative experiments to produce the graph in bottom panel (*n* = 4 for each group). (**E**) Representative images of chick chorioallantoic membrane assay showing embryonated eggs incubated with either control, miR-29, or B7-H3 OE-transfected D283 or D425 cells. (**F**) Bar graphs showing number of blood vessels formed and average tube length per sample. Data assessed using ImageJ (*n* = 3 for each treatment). ** *p* < 0.01, *** *p* < 0.001.

Previous literature has implicated B7-H3 as a pro-angiogenic and miR-29 as an anti-angiogenic molecule [12,25]. To show the angiogenic potential that D283 and D425 cells exhibit, F-actin staining was used to visualize the actin filaments of human umbilical vein endothelial cells (HUVEC’s) alone or co-cultured with MB cells. F-actin staining highlights the microfilament network arrangements, critical components to tumor angiogenesis [30]. The co-cultured HUVEC’s show heightened actin network formation, indicating increased tumor-endothelial interactions (Figure 3A). Additional AlexaFluor staining of B7-H3 (red) shows the presence of B7-H3 prominently in co-culture, indicating the potential role that B7-H3 may play in promoting angiogenesis network formation (Figure 3B).

To further confirm the impact of B7-H3 in angiogenesis and to determine whether the attenuation of B7-H3 has any implications, an in vitro angiogenesis assay was performed (Figure 3C). The angiogenesis tube formation assay using the conditioned media from B7-H3 and miR-29 overexpressing cells (Supplementary Figure S2D,E) was performed. The miR-29 treated cells showed a large reduction in the tube forming ability of HUVEC’s, averaging 100 less rings formed compared to controls for both D283 and D425. The conditioned media obtained from B7-H3 overexpressing (OE) cells showed increased angiogenesis tube formation on average by 50 rings in both cell lines compared to the controls (Figure 3C,D). Interestingly, the tube-forming ability of D283 cells was higher overall when compared to the D425 cells.

The in vivo model of chick chorioallantoic membrane angiogenesis assay was also performed. The effects were similar to the HUVEC assay, with miR-29 overexpressing cells showing poorer vascularization when compared to the controls, and B7-H3 overexpressing cells displaying increased blood vessel formation (Figure 3E). miR-29 cells not only decreased new blood vessel formation, but also reduced established blood vessels and their average tube length while B7-H3 significantly (*p* < 0.001) improved both of these angiogenesis measures in both cell lines (Figure 3F). Collectively, these results indicate that in MB, B7-H3 plays a role in the progression of angiogenesis, and that this effect can be attenuated by miR-29.

### 3.5. Role of sB7-H3 in Angiogenesis

**Figure 4:**
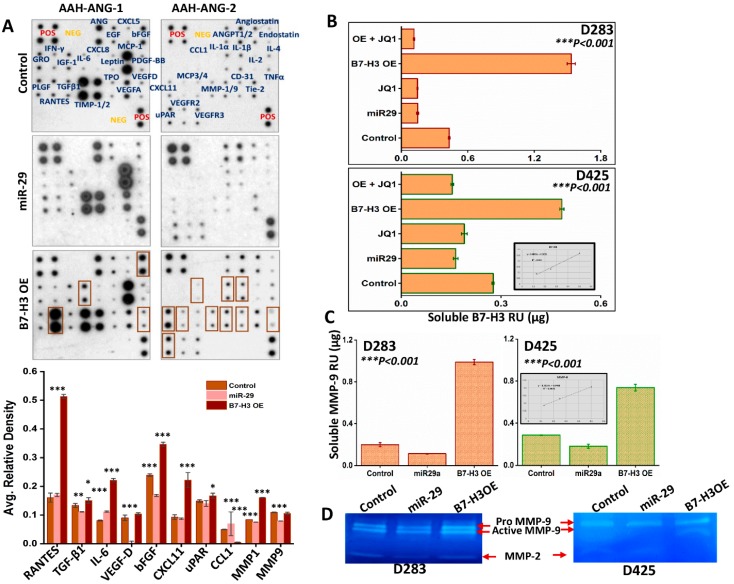
B7-H3 promotes pro-angiogenic molecules released into extracellular medium. (**A**) (**Top**) Human Antibody Angiogenesis Array (RayBiotech, AAH-ANG-1 and 2) incubated with conditioned media from control, miR-29-transfected, or B7-H3 OE-transfected D283 cells, collected 48 h after transfection. (**Bottom**) Densitometry graphs showing difference in average relative density of array proteins (*n* = 4 for each protein). (**B**) Soluble B7-H3 levels from D283 and D425 cell conditioned media assessed by ELISA (*R*^2^ = 0.983) (*n* = 4 for each treatment). (**C**) Soluble MMP-9 levels from D283 and D425 cells transfected with miR-29 plasmid or B7-H3 OE plasmid, 48 h after transfection (*R*^2^ = 0.993) (*n* = 4 for each group). (**D**) MMP-2 and MMP-9 activity levels assessed by gelatin zymography using conditioned media from D283 and D425 cells collected 48 h after transfection. * *p* < 0.05, ** *p* < 0.01, *** *p* < 0.001.

B7-H3 exists in both a membrane-bound and soluble form [31]. The soluble form (sB7-H3) is shown to be functionally active. B7-H3 has been investigated for its role in cancer progression, with researchers finding a relationship between sB7-H3 levels and poorer prognosis across various cancers [32,33]. To further understand the role of sB7-H3 and its association with other proteins in facilitating angiogenesis, we used a human angiogenesis antibody array incubated with conditioned media from D283 control, miR-29- and B7-H3 OE cells. Conditioned media from miR-29-treated cells showed decreased levels of several pro-angiogenic molecules. Conversely, B7-H3 OE showed an increase in pro-angiogenic molecules like IL-6, IL-1, VEGF-D, VEGFR2, CXCL11, uPAR, MCP-3, bFGF, TNF-α and MMP-9, with RANTES (CCL5) showing the largest increase (Figure 4A, top panel). Densitometry analysis confirmed the changes in protein production observed from the arrays (Figure 4A, lower panel). We used enzyme-linked immunosorbent assay (ELISA) to quantify the sB7-H3 levels in various cell treatments. Compared to controls, B7-H3 OE in D283 and D425 yielded higher sB7-H3 levels. However, miR-29 and JQ1-treated- D283 and D425 cells showed lower concentrations of sB7-H3 than controls. These results show that sB7-H3 secretion can be stifled either by direct MYC inhibition or via miR-29 overexpression (Figure 4B). Molecules that appeared to be most differentially expressed by miR-29 or B7-H3 OE treatments, according to the angiogenesis array results, were investigated to further elucidate the specific mechanisms by which sB7-H3 influences angiogenesis. The human angiogenesis array results, and previous studies, indicate that B7-H3 can promote invasion, migration and adhesion by modulating MMP-2/MMP-9 expression [34,35]. We observed from ELISA studies that B7-H3 OE in both D283 and D425 cells upregulated the MMP-9 levels in the conditioned media and miR-29 treatment decreased the MMP-9 levels when compared to controls (Figure 4C). Gelatin zymography assessed MMP-2/9 activity from these treated cells, corroborating the ELISA results (Figure 4D). Together, these results show that B7-H3 OE can upregulate a variety of pro-angiogenic molecules and that the levels of sB7-H3 were correlative with MMP-9 levels and activity in conditioned media. Further, the secretion of B7-H3 can be attenuated via MYC inhibition or miR-29 overexpression in MB cells. 

### 3.6. miR-29 Exhibits Global Anti-Tumor Functions and Promotes STAT1 Activation

**Figure 5:**
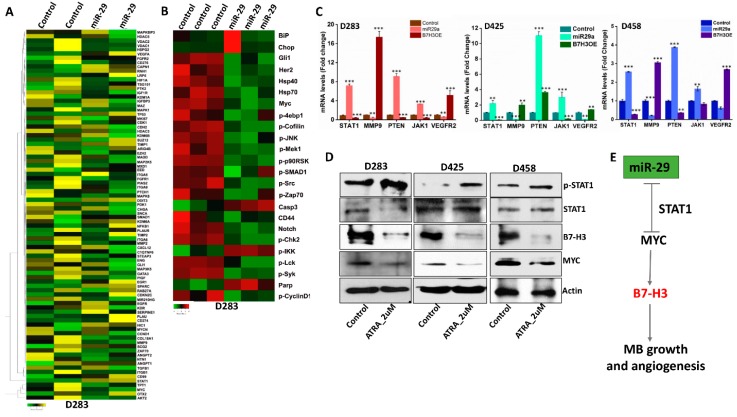
miR-29 overexpression promotes anti-tumor molecules. (**A**) Heat-map showing differentially expressed genes (*p* < 0.05) from miR-29-transfected D283 cells (FPKM = 1). Control cells vs. miR-29 cells in duplicates, increased gene expression (**yellow**) compared to decreased gene expression (**green**). (**B**) Heat-map produced from ActivSignal IPAD data of control and miR-29-transfected D283 cells fixed on a 96-well plate in triplicates. Decreased (**green**) and increased (**red**) protein activity of dysregulated molecules influenced by miR-29 overexpression. (**C**) mRNA levels of 5 genes assessed by RT-PCR (*n* = 4 for each gene) in D283, D425 and D458 transfected cells. (**D**) Protein expression levels of p-STAT1, B7-H3, and MYC in MB cells treated with 2 μM all-trans retinoic acid (ATRA) for 48 h. Actin used as loading control. (**E**) Tumor suppressor role of miR-29 in regulating MYC, B7-H3 expression in MB growth and angiogenesis. * *p* < 0.05, ** *p* < 0.01, *** *p* < 0.001.

To further analyze the global role that miR-29 may play as a tumor suppressor in MB, total RNA from D283 control and cells transfected with miR-29 overexpression plasmid were subjected to RNA-sequencing. The heat-map produced from the RNA-seq results showed a variety of mRNA signatures that were up- or down-regulated as a result of miR-29 overexpression. Most notably, MYC and B7-H3 were downregulated. Interestingly, miR-29 induced a 500-fold upregulation in STAT1 mRNA expression, a molecule implicated in downregulating MYC expression [36,37]. miR-29 downregulated many pro-angiogenic mRNA’s such as VEGFR2, AKT2, MMP2, MMP9, Zap70, MAP2K5 and HIF-1ɑ, further supporting its anti-angiogenesis role in MB (Figure 5A). The RNA-seq data was then integrated into the Upstream Regulator Analysis tool within the Ingenuity Pathway Analysis (IPA) software. The analysis shows a significant transcriptional inhibition of MYC (z = −3.163) and an increase in STAT1 transcriptional activity (z = 2.219) (Supplementary Figure S3A). To further investigate the tumor-suppressive role that miR-29 may play in MB, IPAD analysis was performed, a global signaling network analysis that measures the activity of 70 key proteins involved in more than 20 signaling pathways (https://www.activsignal.com/service/). The results confirmed a decrease in MYC expression along with several major pathway regulators, including p-JNK, p-Cofilin, p-Src, p-Zap70, and p-Mek1 (Ser217/221). miR-29 treatment also increased the activity of Caspase 3, p-IKK and Parp (Figure 5B). Moreover, the integration of RNA-seq data into the IPA tool showed several canonical signaling pathways that were modulated with miR-29 overexpression. Most notably, miR-29 suppressed STAT3, Integrin, VEGF, NF-κB and PI3K/AKT pathways and upregulated the p53, PTEN and JAK/STAT1 signaling pathways (Supplementary Figure S3B). These results collectively suggest that miR-29 has a tumor-suppressive role in MB, in part through the activation of STAT1.

### 3.7. STAT1 Activity Downregulates MYC and B7-H3 Expression in MB Cells

Data from the in silico analysis and RNA-seq indicated that miR-29 promotes STAT1 activation (Figure 5A and Supplementary Figure S3A,B). This is consistent with prior studies indicating STAT1 may play a role as a tumor suppressor, whose function can inhibit the transcription of MYC [36]. To verify the expression of STAT1 and other regulatory targets, we conducted RT-PCR analysis in control, miR-29 and B7-H3 OE Group 3 MB cells. The data showed an increase in STAT1, PTEN and JAK1 in miR-29-treated- D283, D425 and D458 cells, with a concomitant decrease in pro-angiogenic transcripts (MMP-9, VEGFR2). In contrast, B7-H3 OE cells showed an inverse effect on all 5 transcripts (Figure 5C). To further delineate the functional role of STAT1 on B7-H3, MB cells were treated with all-*trans* retinoic acid (ATRA), a known inducer of p-STAT1 [38]. ATRA treatments increased p-STAT1 expression levels; the activation of STAT1 resulted in decreased B7-H3 and MYC protein levels in D283, D425, and D458 cells (Figure 5D). Taken together, miR-29 directly inhibits B7-H3 expression, but it may also induce its tumor-suppressive behavior by upregulating STAT1 activity. STAT1 activation may enhance the anti-tumorigenic effect by further downregulation of MYC expression, stifling MB progression (Figure 5E).

### 3.8. miR-29 in Combination with MYC Inhibition Induces Apoptosis

**Figure 6:**
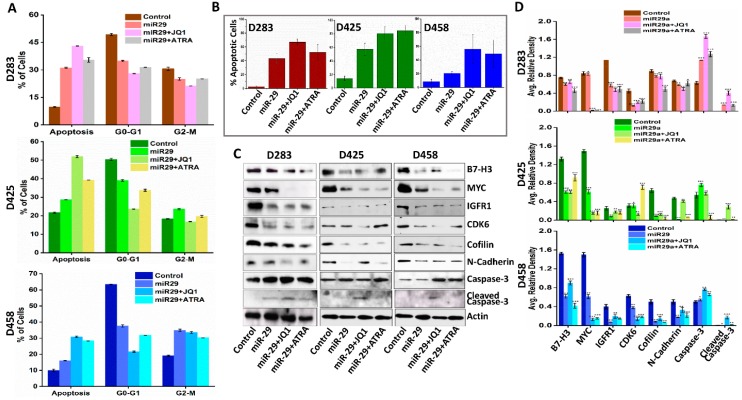
Therapeutic option of combining miR-29 with JQ1 or ATRA in MB. (**A**) FACS analysis of cell cycle progression in D283, D425 and D458 cells 48 h after miR-29 alone and in combination with JQ1 and ATRA treatment. The y axis denotes percentage of total cell count and the x axis represents DNA content. The percentage of cells in the G1 (M2), S (M3) and G2/M (M4) phases of the cell cycle were calculated using CellQuest Pro software. (**B**) Graphs from TUNEL staining assay showing increased apoptosis of combination treatments in D283, D425 and D458 cells (*n* = 3 for each group). Significance calculated compared to control. (**C**) Western blot analysis of whole cell lysates from D283, D425 and D458 control, miR-29, miR-29+JQ1, and miR-29+ATRA cells to determine the expression of molecules associated with cell cycle, apoptosis, and EMT. Actin was used as a loading control. (**D**) Bar graphs from densitometry analysis of Western blots. Band intensity was normalized to each actin band for each sample. * *p* < 0.05, ** *p* < 0.01, *** *p* < 0.001.

A previous clinical trial indicated that miR-29 may have clinical significance as a mediator for enhancing chemo-sensitivity and drug-induced apoptosis [39]. We next validated the therapeutic options of combining miR-29 with MYC inhibition via JQ1 or STAT1 activation via ATRA in order to suppress MB cell growth and progression. The FACS analysis indicated an increased proportion of cells in the G0/G1 phase arrest in either combination treatment when compared to control or miR-29-treated cells (Figure 6A, Supplementary Figure S4A). TUNEL staining confirmed increased apoptotic cells (green) in miR-29 alone and in combination drug-treated cells when compared to controls (Supplementary Figure S4B). Moreover, miR-29 with JQ1 treatment showed the highest proportion of apoptotic cells when compared to control, miR-29 alone or miR-29 with ATRA-treated D283, and D458 cells with either combination treatment showing comparable apoptosis in D425 cells (Figure 6B). Increased apoptosis in the miR-29+JQ1 treatment coincided with increased Caspase-3 activation, confirmed by immunoblot analysis (Figure 6C). Further, miR-29 alone or in combination with JQ1 or ATRA can downregulate the expression of cell proliferation, angiogenesis and EMT-associated molecules such as MYC, CDK6, B7-H3, MMP-9, IGFR1, Cofilin, and N-Cadherin. Densitometry graphs were added to visualize the results from the western blot analyses (Figure 6D). These results provide cumulative evidence that miR-29 may enhance the anti-tumor activity of MYC inhibitors in a synergistic manner. 

## 4. Discussion

Recent data has shown that MB cells disseminate through the bloodstream. The identification of angiogenic drivers and markers in MB can lead to the development of treatments that prevent tumor dissemination, increasing the effectiveness of primary treatments; our data indicates that B7-H3 may be one such target. Investigation into the relationship between B7-H3 and MYC in MB cell lines revealed a transcriptional regulatory relationship between the two molecules. Our results show that in MB cells, MYC can directly control B7-H3 expression and MYC inhibition is an effective method for downregulating B7-H3 in vitro. Further, MYC inhibition appears to upregulate miR-29 expression in MB cells. Unsurprisingly, studies have shown that MYC can regulate a host of miR families including the miR-17-92 cluster [40]. Additionally, miR-29 overexpression reduces B7-H3 expression as corroborated by our data. Previous studies have shown that miR-29 is downregulated across many cancers and overexpression of miR-29 can elicit anti-tumor effects via epigenetic reprogramming, cell apoptosis and inhibition of invasion/migration and angiogenesis [41]. While the relationship between MYC, miR-29 and B7-H3 has been observed in other cancers, our data show the existence of the MYC-B7-H3 regulatory axis in MB that plays a prominent role in regulating angiogenesis. We also showed that miR-29 overexpression attenuated the aforementioned axis, thereby inhibiting MB angiogenesis.

Growing evidence has implicated B7-H3 in the progression of angiogenesis. Studies have shown that increasing B7-H3 expression subsequently increases the expression of MMP-2 in pancreatic cancer and osteosarcoma, consequently increasing the invasive capacity of these cells [35,42]. High expression of B7-H3 was found on surrounding tumor vasculature in kidney, lung, and breast tissue samples [43]. Additionally, inhibition of B7-H3 expression using siRNA in associated endothelial cells decreased the migratory and proliferative capacity of these cells. Evidence from the angiogenesis array and ELISA studies provides further support for this apparent relationship between MMP’s and B7-H3.

Additionally, RNA-seq data shows the downregulation of both MMP-2 and MMP-9 transcripts with miR-29 overexpression. ELISA and zymography data also showed that MMP-2/9 expression and activity is correlated with B7-H3 expression. This implicates B7-H3 as an inducer of angiogenesis via MMP-2 and MMP-9. A previous study observed that B7-H3 knockdown also decreased PI3K/Akt expression, leading to a decrease in STAT3 activity. STAT3 can promote the transcription of MMP2/9, leading the authors to propose PI3K/Akt/STAT3 as mediators of MMP expression via B7-H3 expression, independent of TIMP regulation [34]. Notably, the angiogenesis array data did not show a downregulation of TIMP-1/2, suggesting that B7-H3 promotes MMP-2/9 without reducing their canonical inhibitors. Interestingly, RNA-seq and IPAD data from miR-29-treated D283 cells suggest that p-JNK is downregulated as well, which is known to upregulate MMP-2 expression and promote cancer cell migration [44]. Additionally, IPAD data indicates a decrease in p-Mek1 (Ser217/221) in miR-29-transfected cells, an upregulator of MMP-9 activity in various cancers. Collectively, MYC may upregulate B7-H3, leading to the progression of angiogenesis via MMP-2/MMP-9, and miR-29 may inhibit angiogenesis through B7-H3 and MMP-2/9 inhibition.

Interestingly, RANTES (CCL5) was highly upregulated with B7-H3 OE in the angiogenesis array. Chemokines can act as chemoattractants for a variety of tumor-associated immune cell types, inhibiting the immune response and encouraging a pro-tumorigenic microenvironment. Previous studies have provided evidence for a relationship between B7-H3 expression and chemokine production [45]. Determining the exact nature of this relationship could provide valuable insights into the ways in which B7-H3 influences the tumor microenvironment, and whether combination therapies targeting chemokine receptors on tumor-associated immune cells can improve anti-tumor immune responses in MB patients.

Additionally, IPA results drawn from RNA-seq data indicated a noticeable increase in PTEN signaling. PTEN is considered a tumor suppressor for its role in downregulating PI3K/Akt pathway signaling [46]. The literature investigating the relationship between miR-29 and PTEN has hinted at an epigenetic mechanism for the regulation of PTEN expression via miR-29. DNMT3A methylates the promoter region of *PTEN*, repressing gene expression. DNMT3A can be inhibited by miR-29 [47]. Future investigations into the mechanisms of miR-29 treatment with PTEN stimulators or other PI3K/AKT inhibitors may show synergistic, tumor-suppressive effects.

IPAD data also showed an increase in Caspase-3 and Parp activity in miR-29-transfected cells. Caspase-3 and Parp activity are associated with the progression of cell apoptosis [48,49]. IPAD and IPA results also suggest that miR-29 may inhibit angiogenesis by decreasing p-IKK, the canonical inducer of NF-κB activation [50], indicating an interesting target for future investigations. Additionally, MYC is a well-known regulator of both cell cycle progression and apoptosis [51]. Our studies highlight the increased efficacy in halting tumor cell proliferation which is achieved by using both miR-29 and JQ1 in combination to promote apoptosis. 

The role of the JAK/STAT1 pathway in cancer remains under debate, with evidence showing both anti- and pro-tumorigenic functions across different cancers. The results from this study indicate that STAT1 may contribute to miR-29-mediated tumor inhibition. Some evidence in the literature suggests that STAT1 may be an oncogenic molecule in MYC-independent MB tumors [52], while other studies show how STAT1 activation induces anti-tumor effects in MYC-independent MB [53,54]. Our results reveal that STAT1 activation in MYC-driven MB cells is induced by miR-29, and that up-regulation of this pathway can inhibit MYC and B7-H3. Of note, previous studies have shown that STAT1 activation can promote miR-29 transcription and that miR-29 can decrease tumor cell proliferation by downregulating CDK6, corroborating our western blot data [7,55]. These observations imply an anti-tumor, positive feedback loop between the JAK/STAT1 pathway and miR-29. Overall, these results highlight the utility of targeting MYC^+^ MB tumors using miR-29, STAT1 promoters, and potentially, other promoters of the STAT1 signaling cascade to reduce the tumorigenic effects of MYC dysregulation.

8H9, a monoclonal antibody (mAb) against B7-H3, has been investigated for its therapeutic potential in combating CNS solid tumors [28,56]. Currently, Phase I Clinical Trials are underway utilizing 8H9 (NCT00089245, NCT01502917). The mAb blockade of B7-H3 using 8H9 has shown promising pre-clinical results, and our results indicate a potentially useful combination therapy involving either miR-29 delivery or STAT1 activation. Therapeutic outcomes may be improved by blocking B7-H3-receptor interactions, as well as by ablating B7-H3 expression and activity.

## 5. Conclusion

In summary, this study emphasizes the role that B7-H3 plays in the progression of MYC^+^ MB cells, including angiogenesis. We provide evidence showing that MYC directly regulates the transcription of B7-H3 and may form a regulatory axis with miR-29. In MB cells, angiogenesis can be inhibited with miR-29 overexpression, highlighting the utility of miR-29 as a tumor suppressor in MB. Additionally, miR-29 exhibits anti-tumor effects outside of angiogenesis, including cell cycle inhibition and apoptosis promotion, and appears to elicit synergistic effects with MYC inhibition and STAT1 activation. Confirmation of these results in vivo in future studies will help solidify the targeting of this axis as a viable therapeutic option in MYC^+^ MB. Additionally, further investigation is needed to confirm the efficacy of combination treatments that promote miR-29 and either inhibit MYC activity or promote STAT1 activation in MB, potentially enhancing therapeutic efficacy of treatments targeting B7-H3 in MB tumors. 

## Supplementary Materials

The following are available online at https://www.mdpi.com/2077-0383/8/8/1158/s1, Table S1, List of RT-PCR primer sequences used in study. Table S2, Primers used for ChIP studies. Figure S1, B7-H3, PD-L1, and CTLA-4 mRNA in MB datasets. B7-H3 and MYC mRNA in MB dataset and MB cell lines. JQ1 MTT assay. Figure S2, Western Blot and RT-PCR showing efficacy of B7-H3 OE and miR-29 transfections in MB cells. Figure S3, Graphs of IPA data from miR-29 transfection of D283 cells showing effects on notable signaling pathways. Figure S4, FACS analysis and TUNEL staining of D283, D425, and D458 cell lines showing increased apoptosis when combining miR-29 transfection with JQ1 or ATRA.
