# Supplementary material for: Role of MYC-miR-29-B7-H3 in Medulloblastoma Growth and Angiogenesis
Source: J Clin Med. 2019 Aug 2;8(8):1158. doi: 10.3390/jcm8081158 (PMC6723910; doi:10.3390/jcm8081158)
Supplement: Supplementary file 1 [file jcm-08-01158-s001.pdf]

Supplementary Methods:

MTT Assay

MTT assay (ThermoFisher, Carlsbad, CA, M6494) was carried out using standard protocol (79). Briefly, cells were plated onto 96-well plates in 5 replicates at 20,000 cells/well. Cells were incubated with a range of JQ1 concentrations, from 2 µM, 1 µM, 0.5 µM to 0.125 uM and ATRA concentrations from 4 µM, 2 µM, 1 µM, and 0.5 µM for 24 hrs. Media was removed, and cells were incubated with the MTT reagent (0.5 µg/mL) and incubated at 37ºC for 2 hrs. DMSO was added to each well to dissolve the formazan crystals, after 30 min incubation the absorbance was measured at 550 nm using an xMark microplate spectrophotometer (Benchmark, Bio-Rad, Hercules, CA).

TUNEL assay

TUNEL assay was done using an *in situ* cell death detection kit (Roche Applied Science, Indianapolis, IN) according to the manufacturer's protocol. Briefly, cells on 8-well chambered slides were fixed using 4% paraformaldehyde. Next, cells were incubated with a TUNEL reaction mixture for 60 min at 37° C in a humidified incubator. Before mounting, the slides were washed with PBS and incubated for 5 min with a 1:100 dilution of 4′-6-diamidino-2-phenylindole (DAPI) for nuclear staining and analyzed using confocal microscopy (Olympus BX61 Fluoview) at 40× magnification. The percentage of apoptotic cells observed by TUNEL staining was gathered using ImageJ software.

Table S1: Primer List

B7-H3	Forward	CACCCTACGCTGCTCCTTTTC
	Reverse	CCCTCGTCGGTTACTCGGA
Myc	Forward	GCTGCTTAGACGCTGGATTT

	Reverse	GAGTCGTAGTCGAGGTCATAGTT
miR-29	Forward	CAGAGACTTGAGCATCTGTG
	Reverse	AACCGATTTCAGATGGTGC
STAT1	Forward	CAGCTTGACTCAAAATTCCTGGA
	Reverse	TGAAGATTACGCTTGCTTTTCCT
MMP-9	Forward	AGACCTGGGCAGATTCCAAAC
	Reverse	CGGCAAGTCTTCCGAGTAGT
VEGFR2	Forward	ATTCCTCCCCCGCATCA
	Reverse	GCTCGTTGGCGCACTCTT
PTEN	Forward	TTTGAAGACCATAACCCACCAC
	Reverse	ATTACACCAGTTCGTCCCTTTC
JAK1	Forward	CTTTGCCCTGTATGACGAGAAC
	Reverse	ACCTCATCCGGTAGTGGAGC
U6	Forward	CGCTTCGGCAGCACATATAC
	Reverse	AAAATATGGAACGCTTCACGA
Actin	Forward	ACTCTTCCAGCCTTCCTTCC
	Reverse	TCTCCTTCTGCATCCTGTCG

Table S2: ChIP primers for the *B7-H3 gene promoter*

**Region**	**Forward Primer**	**Reverse Primer**
RI	CACTGTGGTTCTGCCTCACA	CCAGGCAGAGGAAAGAGCAA
RII	ACGTCTGCATGACTGGATCC	GAGTGCTTCCTGTGTGCCAA
RIII	CTCACTGCAACCTCCACCTC	CGGTTTCAGCTGTCCCTGTA
RIV	GACGAGAATCTGTGTGCCCA	TCTTCAGGGACCTGGACCTC
RV	AACCTCATCTGGCAGCTGAC	AATCAGCACTGGGGTTCTGG
RVI	TCACCTCCAGCTCCCTACTC	TGGTGACAGAGCTGTGCG
RVII	TCTGGAAGCCTCTGGGAAGA	CTCTAGGCTACCACCCGAGT
RVIII	CTTCCCCTTGGTTCACCCTC	TGGTCACGTTGCCAGTCAG

Supplementary Figures:

Supplementary Figure 1: B7-H3 and MYC expression in MB subtypes. A. Transcript levels of B7-H3 compared to PD-L1 and CTLA-4 levels in MB datasets from Pfister and Gilbertson. B. B7-H3 and MYC transcript levels from Northcott dataset showing positive correlation across MB subtypes. C. B7-H3 and MYC transcript levels from Cavalli dataset showing positive correlation across MB samples. D. Transcript levels of B7-H3, c-MYC, and n-MYC from RT-PCR of D283 and D425 cells. E. MTT assay showing cell viability of D283 and D425 cells treated with JQ1 (0.5, 1.0, 2.0 μM) for 24 hrs. IC50 value for drug treatments of 1 μM was used in this study.

Supplementary Figure 2: Transfections induce B7-H3 and miR-29 upregulation *in vitro*. A. Immunoblot of D283 cells transfected with shMYC plasmid showing decreased levels of MYC and B7-H3 protein expression. Actin used as loading control. B RT-PCR data showing miR-29 levels in control D283, D425, and D458 cells. C. Immunoblot of D283 cells showing improved inhibition of B7-H3 expression with miR-29 transfection compared to shB7-H3. D. Immunoblot of D283, D425, and D458 cells transfected with B7-H3 OE plasmid observing MYC and B7-H3 protein levels. Actin used as loading control. E. RT-PCR data of D283, D425 and D458 cells transfected with miR-29 or B7-H3 OE plasmid evaluating miR-29 and B7-H3 transcript levels.

Supplementary Figure 3: miR-29 overexpression induces global tumor suppressive effects in MB. A. Upstream Regulator Analysis of RNA-seq data showing probable effect of miR-29 transfection on the activity of relevant signaling pathways. B. Bar graph from IPA of RNA-seq data showing up- (orange) or downregulation (blue) of labelled pathways from miR-29 transfection.

Supplementary Figure 4: miR-29 in combination with JQ1 or ATRA increase MB cell apoptosis *in vitro*. A. Graphs from FACS analysis showing increased concentration of apoptotic cells in miR-29 + JQ1 and miR-29 + ATRA across D283, D425, and D458 cell lines. B. TUNEL staining (green) showing high concentration of apoptotic cells in miR-29 + JQ1 treated D283, D425, and D458 cells when compared to control or miR-29 transfection alone. DAPI (blue) was used to stain cell nuclei.
